# YOLO for early detection and management of Tuta absoluta-induced tomato leaf diseases

**DOI:** 10.3389/fpls.2025.1524630

**Published:** 2025-05-20

**Authors:** Harisu Abdullahi Shehu, Aniebietabasi Ackley, Marvellous Mark, Ofem Ebriba Eteng, Md. Haidar Sharif, Huseyin Kusetogullari

**Affiliations:** ^1^ School of Engineering and Computer Science, Victoria University of Wellington, Wellington, New Zealand; ^2^ School of Architecture and Design, Victoria University of Wellington, Wellington, New Zealand; ^3^ School of Engineering, University of Calabar, Calabar, Cross River, Nigeria; ^4^ Braln Ltd, Port Harcourt, Nigeria; ^5^ Department of Mathematics and Computer Science, St. Mary’s College of Maryland, St. Mary's City, MD, United States; ^6^ Department of Computer Science, Blekinge Institute of Technology, Karlskrona, Sweden

**Keywords:** artificial intelligence in agriculture, dataset, detection, Tuta absoluta, tomato leaf diseases, YOLOv8

## Abstract

The agricultural sector faces persistent threats from plant diseases and pests, with Tuta absoluta posing a severe risk to tomato farming by causing up to 100% crop loss. Timely pest detection is essential for effective intervention, yet traditional methods remain labor-intensive and inefficient. Recent advancements in deep learning offer promising solutions, with YOLOv8 emerging as a leading real-time detection model due to its speed and accuracy, outperforming previous models in on-field deployment. This study focuses on the early detection of Tuta absoluta-induced tomato leaf diseases in Sub-Saharan Africa. The first major contribution is the annotation of a dataset (TomatoEbola), which consists of 326 images and 784 annotations collected from three different farms and is now publicly available. The second key contribution is the proposal of a transfer learning-based approach to evaluate YOLOv8’s performance in detecting Tuta absoluta. Experimental results highlight the model’s effectiveness, with a mean average precision of up to 0.737, outperforming other state-of-the-art methods that achieve less than 0.69, demonstrating its capability for real-world deployment. These findings suggest that AI-driven solutions like YOLOv8 could play a pivotal role in reducing agricultural losses and enhancing food security.

## Graphical Abstract

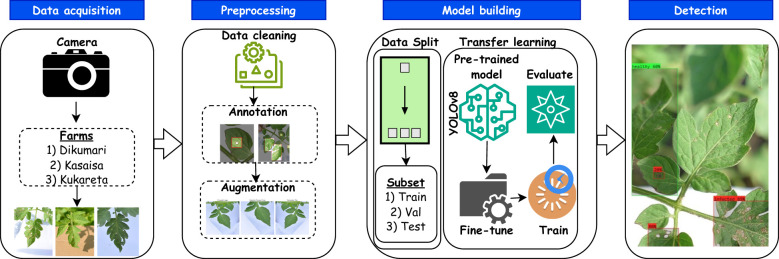

## Highlights

- Annotated the TomatoEbola dataset for precise identification of healthy and diseased tomato leaf regions.
- We developed a YOLOv8-based approach for effective detection of Tuta absoluta-induced diseases in tomato leaves.
- Experimental results highlighted the model’s superior performance and effectiveness.
- The findings demonstrated the model’s real-world applicability in agricultural settings.

## 1 Introduction

In recent years, the agricultural sector has faced significant challenges in ensuring food security due to various factors, including weather and climate conditions, soil degradation, diseases, pests, and environmental pollution (Uygun and Ozguven, 2024). The rise in plant diseases and pest species, exacerbated by climate change, has resulted in increasingly severe crop losses, making it essential to manage these threats to safeguard agricultural productivity (Chakraborty and Newton, 2011). hlClimate change influences pest proliferation by altering temperature and precipitation patterns, thereby extending pest lifecycles and expanding their geographical range, further threatening food security.

Globally, the effects of climate change are being felt across many aspects of life, from heatwaves in Europe and bushfires in Australia to floods caused by Cyclone Gabrielle in New Zealand (Lam and Roy, 2020; Xu et al., 2023). One growing concern is the impact of shifting weather patterns on food security, along with the heightened risk of flooding due to rising sea levels (Lee et al., 2024).

Besides climate change, the complexity of addressing food security has intensified due to factors such as rapid population growth and significant food losses caused by pests. These challenges have made food security one of the most urgent issues facing nations today (Subedi et al., 2023). Pests alone cause billions of dollars in damage annually by destroying fruits and crops, and climate change accelerates their spread by creating more favorable conditions for their survival and reproduction (Agboka et al., 2022; Niassy et al., 2022).

Tomatoes are a major vegetable crop globally, ranking as the world’s top vegetable by output, with annual production exceeding 190 million tons and an average per capita consumption of about 20 kg per year. However, they are highly susceptible to a range of diseases, including the South American tomato pinworm, Tuta absoluta (Meyrick) (Lepidoptera: Gelechiidae), which leads to significant economic losses for growers (Bergougnoux, 2014; Biondi et al., 2018).

A study has found that Tuta absoluta poses a significant threat to tomato crops and can lead to up to 100% crop losses (Desneux et al., 2010) in various regions, including Europe (Campos et al., 2017), Asia (Han et al., 2019), and Africa (Tonnang et al., 2015). Thus, real-time and early detection of Tuta absoluta is crucial for improving pest management decisions and safeguarding tomato yield. Traditional detection of tomato diseases by agricultural personnel is subjective, cumbersome, and time-consuming, leading to a growing demand for innovative, automated, and environmentally friendly pest detection methods (Georgantopoulos et al., 2023).

To bridge this gap, image processing techniques combined with machine learning methods, particularly deep learning algorithms like Convolutional Neural Networks (CNNs) and YOLO (You Only Look Once) models (Redmon et al., 2016), are essential for agricultural pest detection, enabling precise identification and localization of diseases from images (Peng et al., 2023; Badgujar et al., 2024). These detection techniques efficiently process images, making them valuable for real-time detection in plant pest and disease identification.

For example, a study by (Şahin et al., 2023) used YOLOv5 to detect Tuta absoluta larvae and their damage in tomatoes. They collected 1,200 photos of tomato leaves infested by the Tuta absoluta pest to train the YOLOv5 algorithm. Their findings showed that the YOLOv5 algorithm could accurately categorize tomato plant leaves and detect Tuta absoluta larvae and galleries, achieving mean average precision (mAP) rates of 80% and 70-90%, respectively. Similarly, studies by (Malunao et al., 2022; Brucal et al., 2023) used YOLOv3 and YOLOv8 to detect tomato diseases and achieved mean average precision rates of 0.983 and 0.989, respectively. Furthermore, a study by (Ghaderi et al., 2019) used YOLO and VGG16 to detect Tuta absoluta on a dataset gathered in Tanzania. The models achieved a mean average precision of 0.935 with YOLO and a 91.9% accuracy with VGG16.

These studies, along with others conducted in Turkey (Uygun and Ozguven, 2024), Greece (Giakoumoglou et al., 2023), and Tanzania (Mkonyi et al., 2020), have proven these methods effective in various regions where tomato leaf diseases, particularly Tuta absoluta, have caused significant crop damage. However, no study has been conducted to investigate the effectiveness of these methods on Nigerian crops, despite Nigeria being the largest tomato producer in Sub-Saharan Africa and the 14th largest globally (Rwomushana et al., 2019). Tuta absoluta has devastated over 80% of Nigeria’s tomato yields in the past year alone (Tarusikirwa et al., 2020), highlighting an urgent need for region-specific solutions. Additionally, it remains unclear whether models trained on datasets from other countries will generalize effectively to the Nigerian farming environment, given its unique climatic and agricultural conditions.

In a previous study, we created a new dataset of Tuta absoluta-induced tomato leaf disease, termed the TomatoEbola dataset (Shehu et al., 2025). This dataset was collected from three different farms in Nigeria (Dikumari, Kasaisa, and Kukareta farms), each representing different environmental conditions. To evaluate the generalizability of early detection AI methods, we proposed a transfer learning approach using transformers to predict tomato leaf diseases, aiming to improve the model’s adaptability across different datasets. Experimental results demonstrated the effectiveness and generalizability of the proposed approach on both the newly collected TomatoEbola dataset and the widely used PlantVillage (Mohanty et al., 2016) benchmark dataset, achieving an accuracy of up to 99.17%.

In contrast to other benchmark datasets, which are captured in controlled environments with a single leaf per image, the TomatoEbola dataset includes images with multiple leaves in a single frame, which better represents actual conditions in the field. Questions remain about the applicability of classification methods in real-world scenarios for such datasets, especially given that a single frame may contain both healthy and diseased leaves.

However, we know from other studies that YOLO models have proven effective and are capable of detecting both healthy and unhealthy leaves within the same frame (Liu and Wang, 2020a; Liu et al., 2023; Ouf, 2023; Omaye et al., 2024; Wang Y. et al., 2024). This is due to their ability to perform real-time object detection with high accuracy, even in complex and cluttered environments. Therefore, this study investigates the effectiveness of a YOLO model, specifically YOLOv8, in detecting Tuta absoluta-induced tomato leaf diseases on the TomatoEbola dataset, collected in Nigeria. YOLOv8 was chosen due to its improved architecture, faster inference speed, and enhanced accuracy for detecting small objects, such as pest larvae on leaves.

The main contributions of this work are as follows:

1. Annotate the TomatoEbola dataset with precise bounding boxes, effectively delineating healthy and unhealthy regions on tomato leaves to facilitate robust training and evaluation.
2. Propose a transfer learning approach for detection Tuta absoluta-induced tomato leaf diseases utilizing the advanced capabilities of the YOLOv8 model, tailored for high accuracy in challenging agricultural environments.
3. Conduct comprehensive experimental evaluations to demonstrate the model’s effectiveness, emphasizing critical metrics such as real-time detection speed and overall accuracy in diverse conditions.
4. Emphasize the potential of AI-driven solutions to significantly reduce agricultural losses attributed to pests like Tuta absoluta, paving the way for sustainable farming practices and enhanced crop management.

This research advances the field of plant disease detection by utilizing deep learning-based object identification algorithms to promote more effective and sustainable management of tomato leaf diseases.

The remainder of the paper is structured as follows: Section 2 provides an overview of the history, lifecycle, and impact of Tuta absoluta on tomato plants. It also discusses the technical barriers hindering early detection of tomato leaf diseases, object detection techniques, and the YOLO series, highlighting their advantages and explaining the choice of YOLOv8 over other models. Additionally, recent research on plant leaf disease detection using state-of-the-art methods is reviewed. Section 3 describes the study area, image acquisition procedure, dataset creation and annotation process, dataset characteristics, and augmentation techniques used to enhance data diversity. The section concludes by introducing the study methodology. Section 4 presents the experimental work, including the hardware setup, results obtained by the proposed method, and comparisons with relevant studies and state-of-the-art techniques. Section 5 discusses the results in detail, identifies limitations, and suggests directions for future research. Finally, Section 6 concludes the paper.

## 2 Background

### 2.1 Tuta absoluta tomato leaf diseases

Tuta absoluta was originally described as Phthorimaea absoluta by Meyrick in 1917 from specimens found in Huancayo, Peru, and has been reclassified several times under genera such as Gnorimoschema, Scrobipalpula, and Scrobipalpuloides. It was officially renamed Tuta absoluta in 1994 (Biondi et al., 2018), and it is commonly known as the South American tomato pinworm (Crespo-Pérez et al., 2015).

The feeding behavior of Tuta absoluta makes early infestation detection difficult, often resulting in severe damage to young plants. Additionally, its feeding on fruits diminishes their appearance, raising the expenses of post-harvest sorting before they can be marketed (De Castro et al., 2013). Tuta absoluta is known for its ability to feed, survive, and reproduce successfully on a diverse range of host plants (Arnó et al., 2019), including tomato (Sylla et al., 2019). It has a life cycle of 26 to 75 days and developmental thresholds between 14 °C and 36 °C, within a humidity range of 32% to 72% (Martins et al., 2016).

During the 1960s, Tuta absoluta was reported to have spread from the central highlands of Peru to several other Latin American countries (Campos et al., 2017; Biondi et al., 2018). Since then, it has been detected in numerous countries, including Spain in 2006 (Aigbedion-Atalor et al., 2020), Italy in 2008 (Speranza and Sannino, 2012), South Africa in 2016 (Son et al., 2017), and Nigeria in 2010 (Tarusikirwa et al., 2020), as well as other tomato-growing regions along the Mediterranean coast. Please refer to (de Campos et al., 2021) for more discussion on the biology, lifecycle, and global spread of Tuta absoluta.

The Tuta absoluta pest poses a significant threat to tomato production (Desneux et al., 2011). For instance, recent studies have revealed that Tuta absoluta has damaged over 80% of tomato crops in Nigeria (Tarusikirwa et al., 2020). However, while the pest’s impact has been extensively studied in other countries, including Greece, Tanzania, and Turkey (see **Table 1**), there remains a gap in research regarding the use of AI methods in detecting Tuta absoluta-induced tomato leaf diseases. No study has yet been conducted to investigate the effectiveness of AI approaches for this purpose based on a dataset collected from Nigeria, the largest tomato producer is Sub-Sahara Africa, where the prevalence and severity of Tuta absoluta infestations continue to rise, impacting the livelihoods of local farmers. Therefore, this study aims to evaluate the capability of AI methods, specifically a YOLO model, in detecting tomato leaf diseases caused by Tuta absoluta, based on a dataset collected in Nigeria.

**Table 1:** Recent studies have been conducted on detecting Tuta absoluta tomato plant leaf disease.

Reference	Dataset	Collection Mode	Method	Parameters	Data Grouping	Country	Performance
(Mkonyi et al., 2020)	Collected	Photography	VGG16	Epochs = 1000 Batch size = 8 Optimizer = sigmoid lr = 1e-5 Dropout = 0.5 Momentum = 0.9 Early stopping = 50 epochs	85-15 split	Tanzania	91.9%
(Giakoumoglou et al., 2023)	Collected	Photography	Faster R-CNN	Epochs = 20000 Batch size = 2 Optimizer = sigmoid lr = 1e-3 Momentum = 0.9	60-40 split	Greece	mAP = 0.58
(Loyani et al., 2021)	Collected	Photography	CNN	Epochs = 200 Batch size = 1 lr = 1e-3 Decay = 1e-4 Momentum = 0.9	80-20 split	Tanzania	85.67%
(Uygun and Ozguven, 2024)	Collected	Photography	YOLO	Epochs = 100 Batch size = 8 lr = 1e-2 Optimizer = sigmoid Decay = 5e-4 Momentum = 0.937	70-30 split	Turkey	mAP = 0.935
(Bütüner et al., 2024)	Collected	Photography	Decision Tree	**-**	80-20 split	Turkey	98.7%
(Loyani and Machuve, 2021)	Collected	Photography	CNN	Epochs = 200 lr = 1e-2 Optimizer = Adam	80-20 split	Tanzania	70%
(Loyani, 2024)	Collected	Photography	CNN	Epochs = 200 lr = 1e-3	80-20 split	Tanzania	mAP = 0.857

Note that lr, learning rate; mAP, mean average precision. All studies used accuracy as an evaluation metric, except for those that explicitly report mAP.

### 2.2 Technical barriers

Early detection of Tuta absoluta infestations in tomato plants is hindered by several technical challenges, including the small size of lesions and the ambiguity of symptoms. The initial damage appears as minor mines or blotches on leaves, often making it difficult to distinguish from other plant stressors (Rwomushana et al., 2019).

The larvae’s feeding habits further complicate detection, as they tunnel inside leaves, stems, and fruits while leaving the epidermis intact. This concealed activity makes infestations less noticeable in the early stages, leading to potential delays in diagnosis (Rwomushana et al., 2019).

Moreover, the diversity in symptoms across different tomato leaf diseases adds another layer of complexity. Symptoms vary in size and appearance, and certain diseases, such as tomato leaf mold and late blight, exhibit lesions that are highly sensitive to environmental factors like light exposure, making them harder to differentiate (Wang Y. et al., 2024).

These challenges are particularly relevant to Tuta absoluta-induced damage, which often presents with overlapping visual symptoms. Distinguishing Tuta absoluta infestations from fungal infections or nutrient deficiencies can be difficult, increasing the risk of misclassification in automated detection systems. This underscores the need for carefully curated training datasets that reflect real-world farming conditions. Factors such as humidity, temperature, and light exposure must be considered when collecting data, as they can influence symptom expression. Thus, ensuring that AI models are trained on diverse, high-quality data is critical for improving their ability to accurately detect and differentiate subtle variations in tomato plant diseases.

### 2.3 Object detection

Object detection is a computer vision technique that identifies and locates objects within images or videos (Zhao et al., 2019). Unlike image classification, it provides object positions with bounding boxes, enabling the detection of multiple objects simultaneously. Using deep learning algorithms, often Convolutional Neural Networks (CNNs), object detection predicts the location and class of each object. It is vital in applications like autonomous driving (Qian et al., 2022), surveillance (Kumar et al., 2020), medical imaging (Ragab et al., 2024), and agriculture (Badgujar et al., 2024).

Popular models include Faster R-CNN (Girshick, 2015) for high accuracy, SSD (Liu et al., 2016) for real-time processing, and RetinaNet (Lin et al., 2017) for handling class imbalance. In contrast, YOLO (You Only Look Once) (Redmon et al., 2016) series, including versions from YOLOv1 to YOLOv12 (see Section 2.4), are widely used for their speed and versatility. Their ability to process images in a single pass while maintaining competitive accuracy makes them ideal for detecting Tuta absoluta-induced tomato diseases, where rapid identification is crucial for timely intervention.

### 2.4 You only look once model

**Figure 1:**
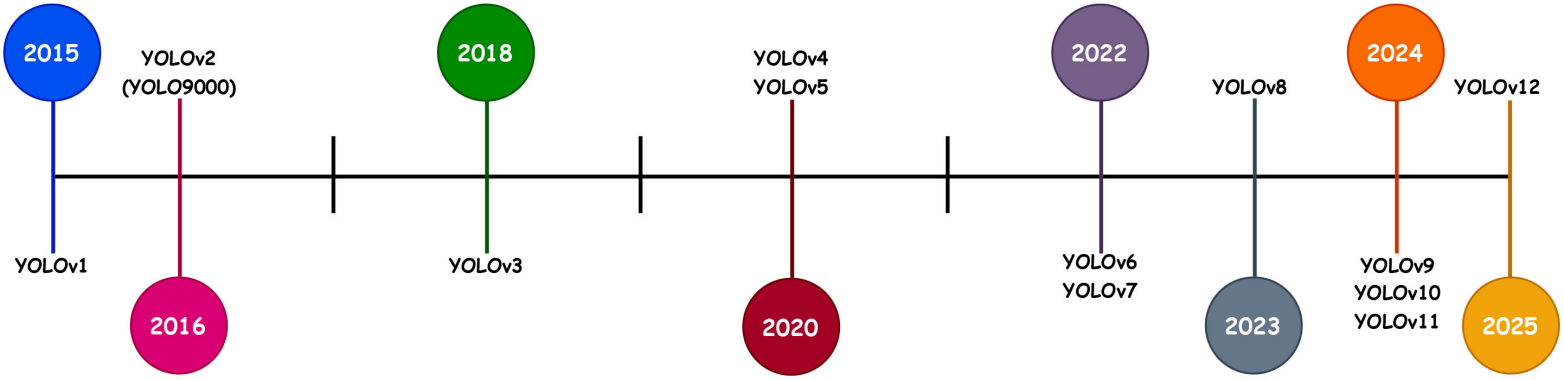
Timeline showing the release of YOLO versions.

YOLO (You Only Look Once) (Redmon et al., 2016) is widely used for real-time object detection, including in plant pest and disease detection. **Figure 1** demonstrates the timeline for the release of different YOLO versions. Since its introduction, the YOLO family has progressed through several iterations, with each version advancing from the previous ones to overcome limitations and improve performance (Terven et al., 2023).

Its versions, such as YOLOv2 (Redmon and Farhadi, 2017), YOLOv3 (Redmon, 2018), YOLOv4 (Bochkovskiy et al., 2020), and YOLOv5 (Jocher, 2020), have advanced in accuracy and speed, with YOLOv3 introducing multi-scale prediction and YOLOv4 enhancing GPU optimization. YOLOv6 (Li et al., 2022) focuses on edge device deployment, while YOLOv7 (Wang et al., 2023) refines architecture for speed and generalization. YOLOv8 (Solawetz and Francesco, 2023) improves object scaling and interpretability, crucial for detecting small or subtle disease symptoms on tomato leaves (Ahmed and Abd-Elkawy, 2024; Ma et al., 2024), making it ideal for this study. YOLOv9 (Wang C-Y. et al., 2024) and YOLOv10 (Wang A. et al., 2024) further enhance performance with neural architecture search and transformer modules. YOLOv11 (Ultralytics, 2024) optimizes speed and accuracy with fewer parameters, and finally, YOLOv12 (Tian et al., 2025) introduced an attention-centric framework, outperforming earlier versions in terms of performance.

YOLOv8’s auto-scaling and improved interpretability make it the model of choice for agricultural disease management (Quach et al., 2024).

### 2.5 State-of-the-art methods in plant disease detection

Deep learning, as the state-of-the-art method in plant leaf disease detection, has achieved promising accuracy and robustness in various real-world applications. For instance, the transformer architecture has been modified to support computer vision tasks through the Vision Transformer (ViT) (Thakur et al., 2021). Similarly, deep Convolutional Neural Network (CNN) approaches like GoogleNet, ResNet, VGG, Inception, and EfficientNet have all been applied to various plant disease detection tasks (Ferentinos, 2018; Shaheed et al., 2023).

Swami et al. (2022) proposed a VGG architecture to detect tomato diseases by analyzing the leaves through a combination of transfer learning on the Plant Village dataset. Similarly, Shi et al. (2022) applied transfer learning to retrain an EfficientNetV2 model to detect tomato diseases. The proposed model not only achieved state-of-the-art performance but was also deployed in the field through a hosted instance in the cloud and an integrated Android application for real-time disease identification and monitoring.

In addition, object detection frameworks like YOLO and Faster R-CNN have also been applied to plant disease detection tasks. These models are suitable for real-time object detection, capable of processing images at high speeds while maintaining accuracy.

For instance, Yu et al. (2023) modified the CNN architecture in YOLOv5 to shrink the parameters, introduced an attention mechanism, and modified the loss function to detect tomato diseases with improved model speed and efficiency. The Faster R-CNN model has been proposed to detect tomato leaf diseases from images, achieving a mean average precision of 0.58, which was considered reasonable due to the complexity of the data and the challenges of implementing a real-time study (Giakoumoglou et al., 2023).

However, these state-of-the-art methods have their limitations. For instance, CNN-based models often require large datasets for optimal performance, making them less effective in cases with limited labeled data. Additionally, transformer-based models like ViT, while effective, demand high computational resources, limiting their deployment in real-time applications. Object detection models such as YOLOv5 and Faster R-CNN, although efficient, may struggle with accurately detecting subtle lesions due to variations in disease appearance and environmental factors. To address these limitations, this study proposes a transfer learning approach using YOLOv8. This approach leverages pre-trained weights to enhance feature extraction, improving detection accuracy while maintaining computational efficiency. Additionally, the model is optimized for real-time deployment, ensuring practical applicability in field conditions.

## 3 Materials and methods

### 3.1 Data collection

This section details the collection of tomato leaf images from three farms in Yobe State, Northern Nigeria, emphasizing the geographical context and the creation of the dataset.

#### 3.1.1 Study area

**Figure 2:**
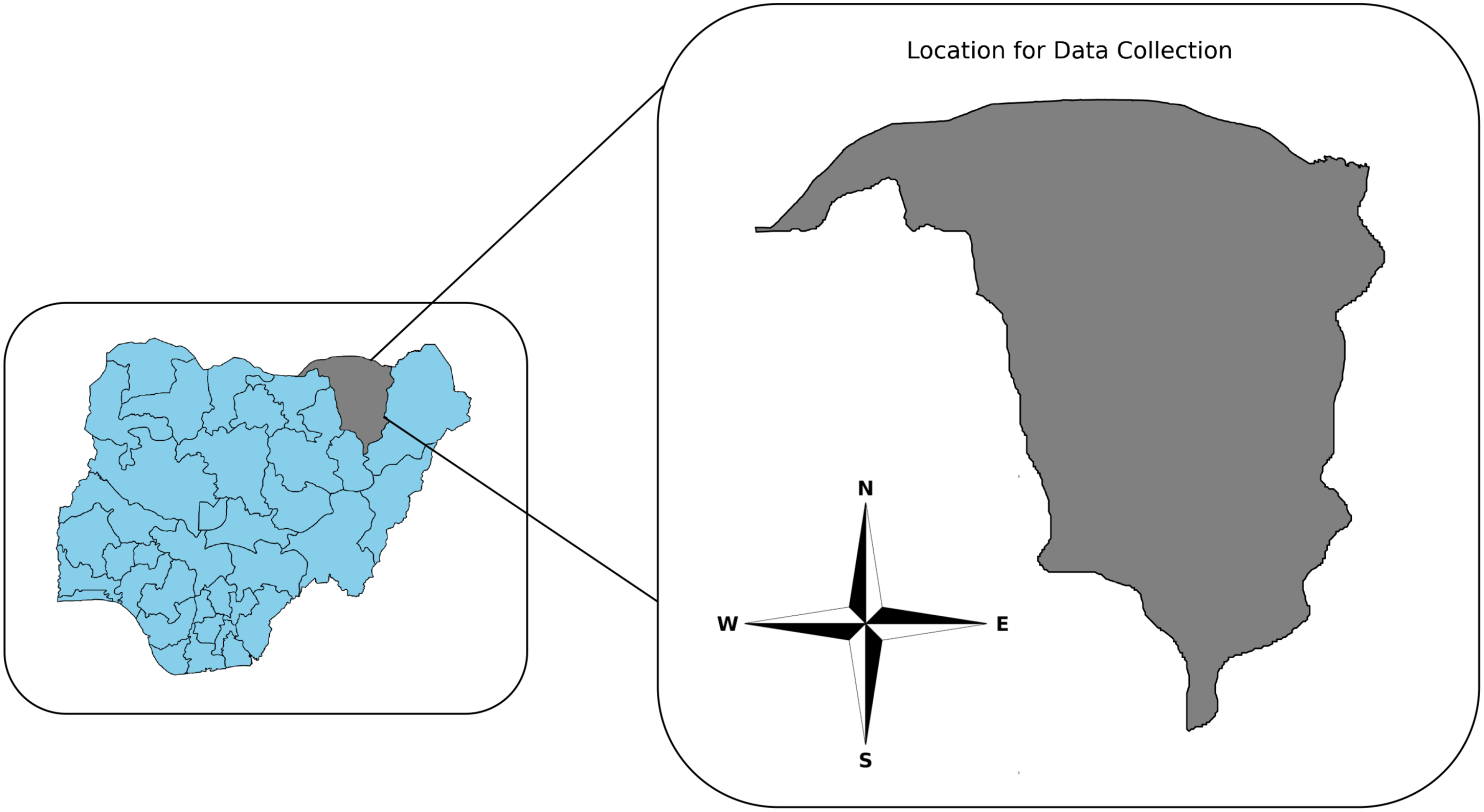
Map of the study location highlighting Yobe State, Nigeria. The cardinal directions (N, S, E, and W) are indicated on the map to assist in orientation.

Tomato leaf images were obtained from three prominent farms situated in Yobe State, Northern Nigeria. The location of the data collection is illustrated in **Figure 2**. These images were gathered during the early rainy season, starting with farms in the Dikumari and Kukareta districts in late May 2024, followed by collection from a farm in the Kasaisa district in mid-June 2024.

**Figure 3:**
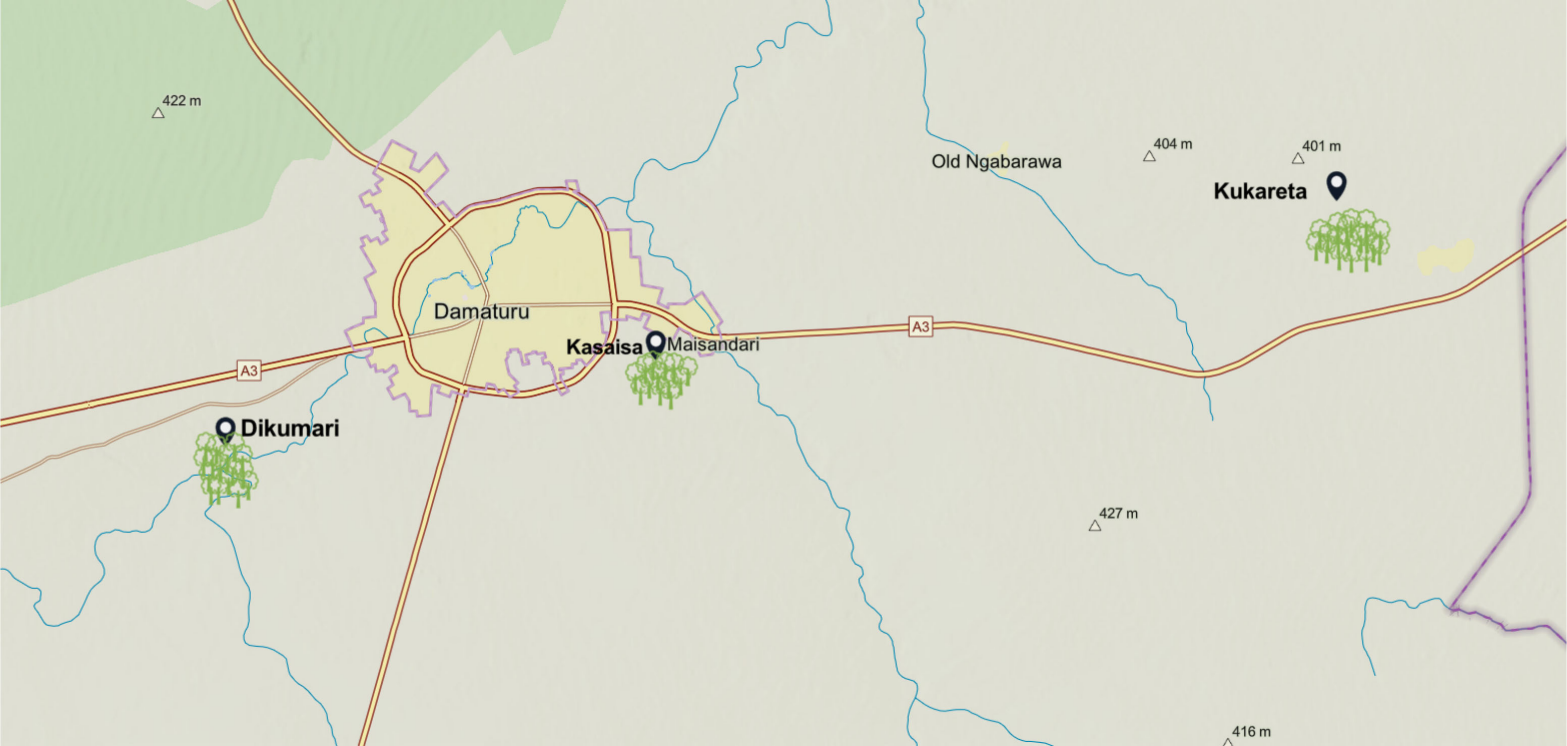
Map showing the locations of the three farms in Yobe State, Nigeria.

The approximate geographical locations of these farms are depicted in **Figure 3**, as sourced from ArcGIS^^1^^.

#### 3.1.2 Image acquisition and dataset building

The study adhered to established protocols and guidelines, emphasizing the capture of images of both healthy tomato leaves and those affected by varying degrees of infestation, particularly focusing on leaves infested with Tuta Absoluta. The images were captured based on expert evaluation of symptoms, ensuring that the selection process accurately represented the various stages of infestation. A Nikon D610 camera, featuring a 24.3MP FX-format CMOS sensor and capable of continuous shooting at 6 frames per second, was used with a 50mm lens positioned 1.3 meters above the leaves during image capture. To maintain image quality, the camera was kept at a consistent distance from the leaves, and images were taken around midday in optimal natural lighting conditions, utilizing appropriate settings to minimize noise.

From the naturally captured images of diseased leaves, 326 images representing various health conditions of the tomato leaves were selected. This included 174 images of leaves with different levels of infestation and 152 images of healthy leaves collected from the three farms. This selection led to the creation of a diverse dataset of 326 images that include both healthy and diseased tomato leaves in their natural setting. The newly formed dataset, referred to as the TomatoEbola dataset, serves as a robust resource for further analysis and model training.

Further details about the dataset can be found in Section 3.2.

### 3.2 TomatoEbola dataset

This section provides a description, annotation, augmentation, and characteristics of the TomatoEbola datasets captured from the three farms.

#### 3.2.1 Description

The TomatoEbola dataset is a newly curated dataset designed to address the specific challenges posed by tomato leaf diseases, particularly the Tomato Leafminer Tuta absoluta, which is prevalent in certain regions. This dataset, collected from three farms in Yobe State, Nigeria (see **Figure 3**), includes a comprehensive collection of images depicting both healthy and infected tomato leaves. Focusing on region-specific data, the TomatoEbola dataset aims to complement existing datasets, providing valuable information that enhances the accuracy and generalizability of disease prediction models.

**Figure 4:**
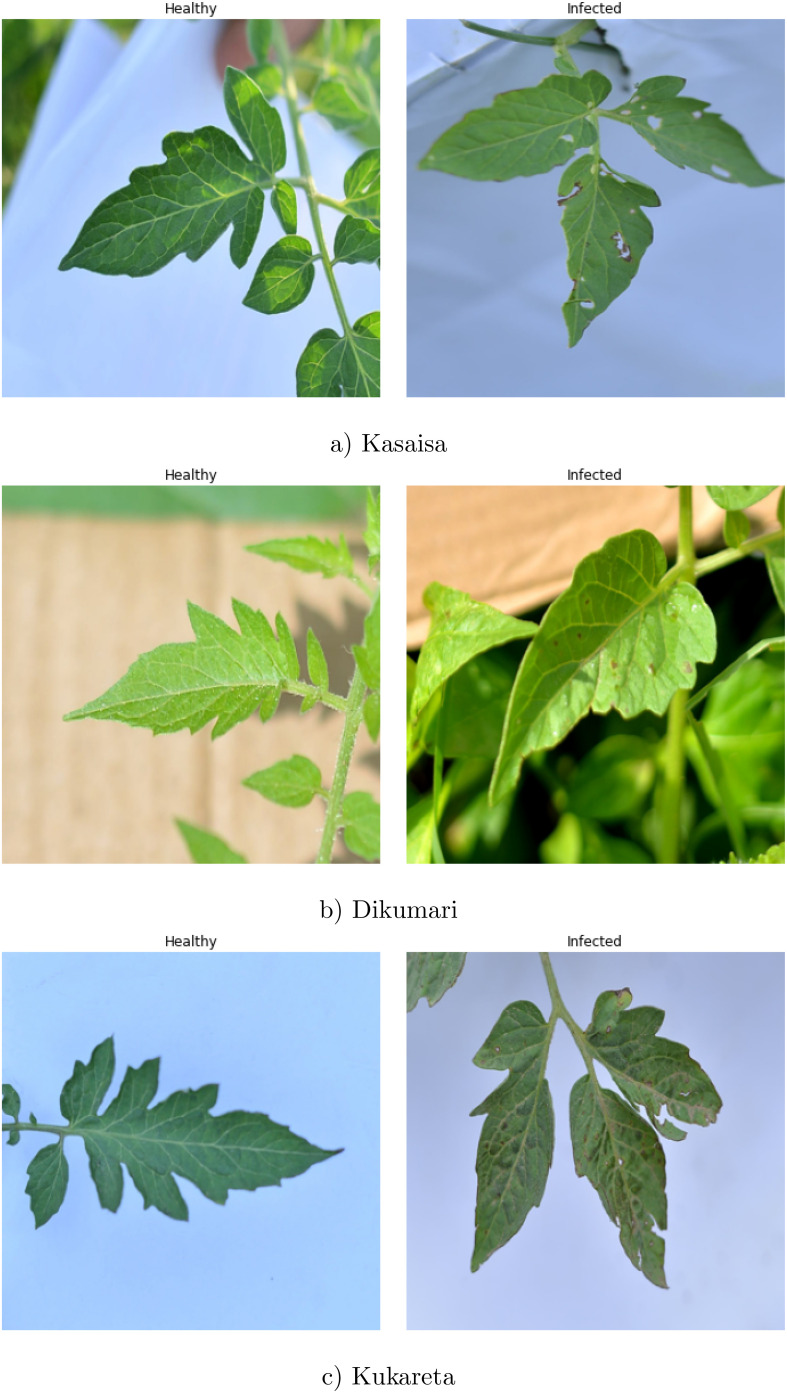
Examples of tomato leaves from the TomatoEbola dataset. Each set contains healthy and unhealthy images from **(a)** Kasaisa, **(b)** Dikumari, and **(c)** Kukareta farms.

**Figure 4** depicts sample images from the TomatoEbola dataset. A detailed breakdown of the images from the TomatoEbola dataset can be found on **Table 2**.

**Table 2:** Summary of the Kasaisa, Dikumari, and Kukareta Datasets.

Farm		Images	Annotations			Image Properties	
	Total	Avg. Annotations	Total	Healthy	Infected	Size (megapixel)	Median Ratio
Kasaisa	122	2.6	319	138	181	0.62	667x906
Dikumari	101	2.1	217	117	100	2.24	1345x1673
Kukareta	103	2.4	248	164	84	0.87	929x943

The table provides details on the number of images, total annotations, average annotations per image, image size, median image ratio, and the distribution of healthy and infected annotations for each dataset.

#### 3.2.2 Annotation

All annotations for the datasets were performed using the Roboflow software (Roboflow, 2024a). This tool facilitated the labeling process by allowing precise and efficient annotation of image data. **Supplementary Figure 1** provides a visualization of the statistical analysis of the bounding box labels.

**Table 2** provides a summary of the total number of annotations performed on each class of images within the datasets. Each image was manually labeled by experts to ensure accurate identification of healthy and infected leaves. However, the annotation of subtle lesions required additional scrutiny to maintain consistency across the dataset.

It is worth noting that no augmentation techniques (see 3.2.3) were applied during the creation of these datasets. Consequently, all labeled images are original and were captured directly from their respective environments. This ensures that the dataset reflects real-world conditions without additional modifications, preserving its authenticity. Researchers who wish to train their models may choose to use augmentation to increase the diversity of the dataset.

#### 3.2.3 Augmentation

The augmentation pipeline consists of several image transformations de-signed to enhance the diversity and robustness of the training dataset.

Augmentation techniques were applied to increase both the size and diversity of the training data. Hue, saturation, and value (HSV) Shift modifies the hue, saturation, and value of the image to simulate different lighting conditions. Translation shifts the image by 10% of its dimensions, providing variation in positioning. Scaling reduces the image size by half while maintaining its aspect ratio through resizing and padding. Horizontal Flip creates a mirrored version of the image, offering an alternative perspective. Mosaic combines four images into a single composite image, preserving the contextual relationships among them. Random Erasing randomly removes a significant portion (40%) of the image to introduce occlusions, encouraging the model to focus on other features. Lastly, RandAugment randomly selects and applies an augmentation technique, which, in this case, is a vertical flip of the image, introducing variability and preventing overfitting during training.

Together, these methods enrich the dataset, enabling the model to generalize better to unseen data.

#### 3.2.4 Characteristics

**Table 2** summarizes the key characteristics of the Kasaisa, Dikumari, and Kukareta datasets. Each dataset varies in the number of images, annotation count, and median image ratios, reflecting differences in data complexity and class distribution. Kasaisa has the highest average annotations per image, while Dikumari has the largest median image ratio and image size. Kukareta shows a balanced number of annotations but has a smaller image size compared to Dikumari, providing a diverse representation of healthy and infected annotations across the datasets.

**Figure 5:**
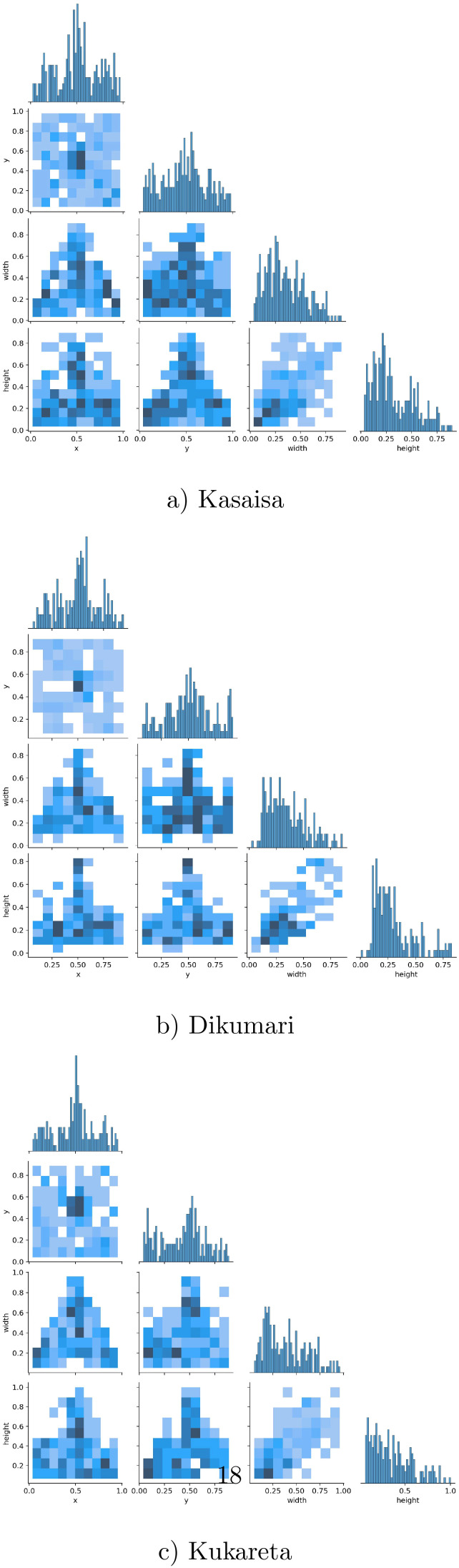
Correlogram distribution graphs of the bounding box labels of the data from **(a)** Kasaisa, **(b)** Dikumari, and **(c)** Kukareta farms.

**Figure 5** shows the correlogram distribution graphs of the bounding box labels from the data collected at (a) Kasaisa, (b) Dikumari, and (c) Kukareta farms. These graphs illustrate the spatial distribution and density of disease occurrences within the images from each farm, highlighting potential differences in disease patterns and spread across the datasets. The visualization provides insights into how diseases manifest differently at each site, which could impact the development and evaluation of detection models.

### 3.3 YOLOv8

YOLOv8 is a state-of-the-art deep learning model designed for various computer vision tasks, including object detection, segmentation, pose estimation, tracking, and classification (see Section 2). It offers five scaled versions tailored to different computational and performance needs: YOLOv8n (nano), YOLOv8s (small), YOLOv8m (medium), YOLOv8l (large), and YOLOv8x (extra-large). Each version balances accuracy and speed, allowing researchers to select the most suitable model for their specific application.

**Figure 6:**
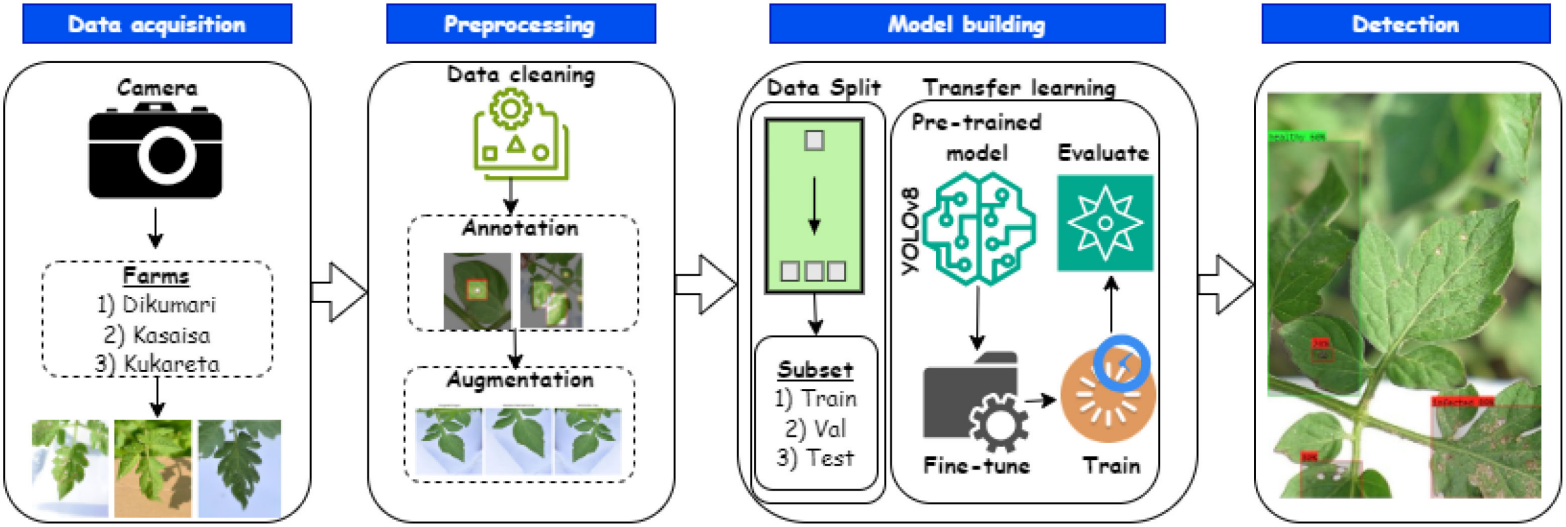
Overview of an end-to-end method for predicting Tuta absoluta in tomato leaves using YOLOv8.

**Figure 6** presents an overview of the approach for predicting tomato leaf diseases. In this study, we utilized the YOLOv8l (large) version. This choice was primarily based on empirical findings (see Section 4.4) demonstrating that YOLOv8l is particularly well-suited for detecting tomato leaf diseases from the TomatoEbola dataset, characterized by a small sample size. Thus, its efficiency, providing a balance between precision and lower computational requirements, makes it ideal for tasks such as this, requiring real-time object detection.

YOLOv8l was employed to perform object detection by predicting bounding boxes and class probabilities in a single forward pass through the network. The procedure involves dividing the input image into a grid of cells, each responsible for detecting objects within its region. For each cell, the model predicts bounding boxes, class probabilities, and confidence scores. The model optimizes a multi-part loss function L that combines classification loss $L_{cls}$ , localization loss $L_{loc}$ , and objectness loss $L_{obj}$ , as shown in Equation 1.


(1) $$L=\lambda_{cls}L_{cls}+\lambda_{loc}L_{loc}+\lambda_{obj}L_{obj}$$

where $\lambda_{cls}$ , $\lambda_{loc}$ , and $\lambda_{obj}$ are the weights balancing the contributions of each component. The classification loss $L_{cls}$ measures the accuracy of class predictions, the localization loss $L_{loc}$ assesses the precision of the predicted bounding box coordinates, and the objectness loss $L_{obj}$ evaluates the likelihood that the predicted boxes contain objects. This combination of losses ensures that the model not only learns to classify objects accurately but also improves the precision of bounding boxes, crucial for detecting the subtle symptoms of tomato leaf diseases with high accuracy.

**Figure 7:**
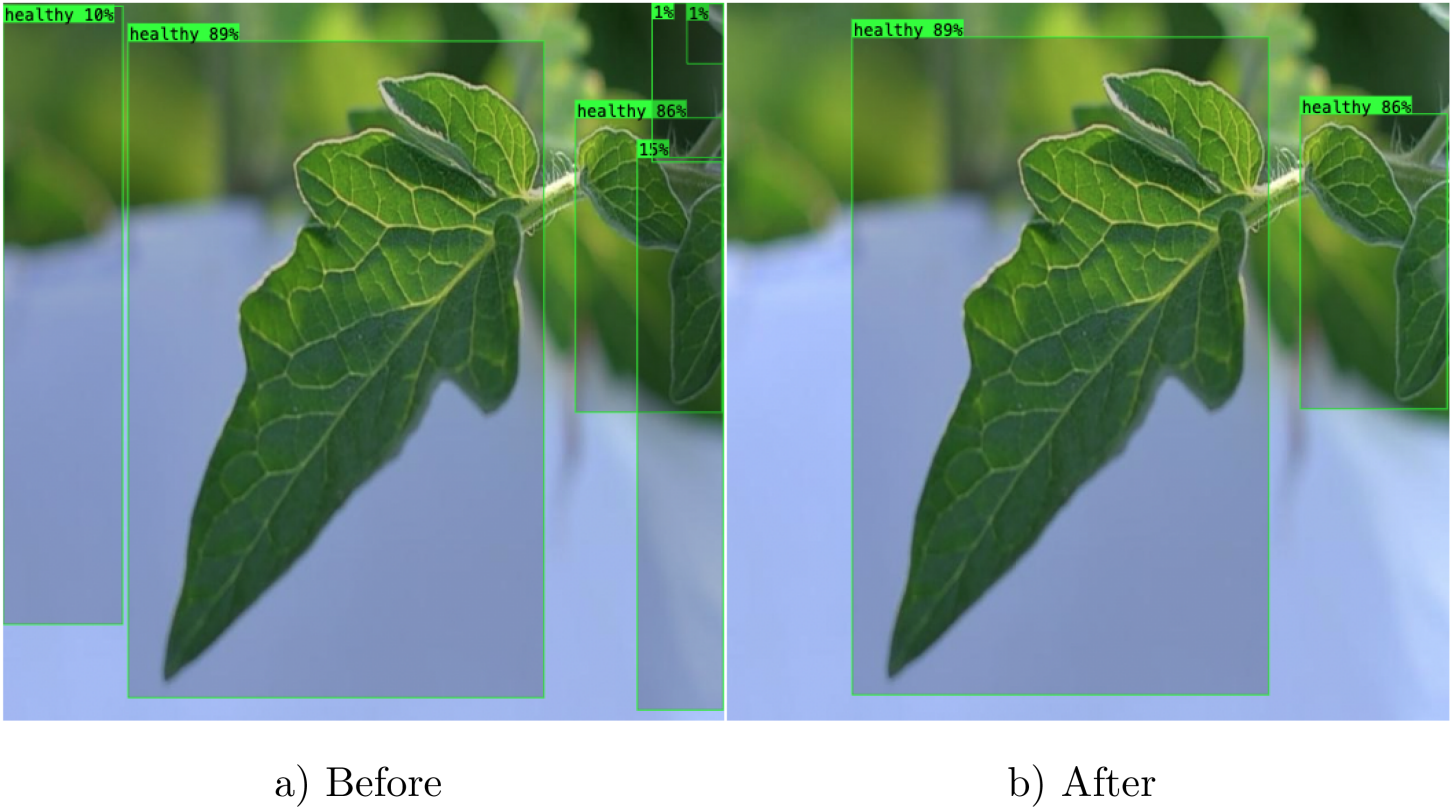
Exemplar predictions by the YOLOv8l model **(a)** before and **(b)** after filtering irrelevant bounding boxes using the Non-Maximum Suppression algorithm.

Finally, we filtered redundant and irrelevant bounding boxes using the Non-Maximum Suppression (NMS) algorithm with a threshold (*T*) value of 0.5 (see **Algorithm 1**). Bounding boxes with confidence scores below this threshold were removed, retaining only those with scores above *T* in the final output (see **Figure 7**).

Algorithm 1 **Non-Maximum Suppression Algorithm**

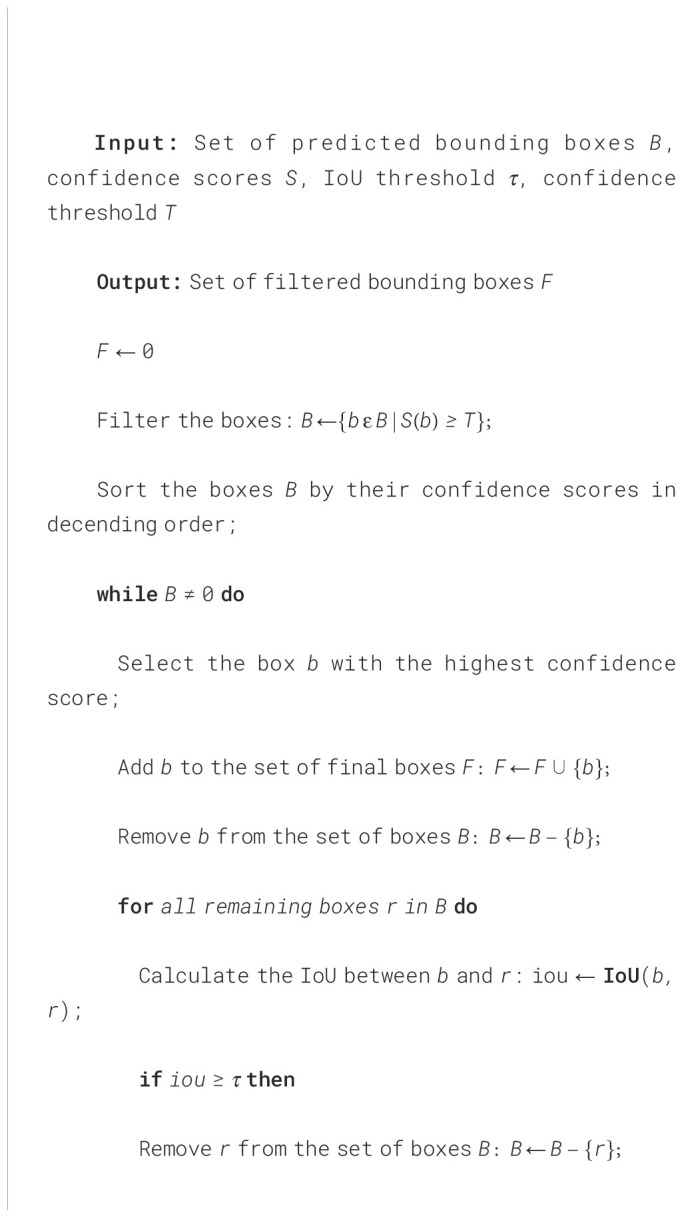

## 4 Experimental work

### 4.1 Hardware specification

A MacBook M1 laptop with 16 GB RAM, an M1 GPU, and a 512 GB SSD was used for these experiments.

### 4.2 Parameter settings

A number of different parameters, such as varying epochs, learning rates, and batch sizes, have been experimented with during the model training phase. Among these, the parameters listed in **Table 3** provided the best accuracy results.

**Table 3:** Summary of training parameters used for model optimization.

Parameter	Value
Epochs	100
Batch Size	16
Image Size	640 x 640
Workers	8
Learning Rate (lr)	0.01
Momentum	0.937
Weight Decay	0.0005
Warmup Epochs	3.0
Warmup Momentum	0.8
Warmup Bias Learning Rate	0.1

The table lists key hyperparameters that were used during the training process to enhance model performance.

### 4.3 Evaluation metrics

The evaluation metrics used are precision, recall, and mean average precision (mAP). These metrics provide insights into the effectiveness of the model in terms of its accuracy and ability to detect the desired classes.

#### 4.3.1 Precision

Precision measures the proportion of true positive detections among all positive detections made by the model. The formula for computing recall is given in Equation 2.


(2) $$\text{Precision}=\frac{TP}{TP+FP}$$

where *TP* represents the number of true positives and *FP* represents the number of false positives.

#### 4.3.2 Recall

Recall measures the proportion of true positive detections among all actual positive instances. The formula for computing recall is given in Equation 3.


(3) $$\text{Recall}=\frac{TP}{TP+FN}$$

where *TP* represents the number of true positives and *FN* represents the number of false negatives.

#### 4.3.3 Mean average precision

Mean Average Precision, as presented in Equation 4, is the average of the average precision scores for each class. Average Precision (AP) for a class is calculated by taking the area under the precision-recall curve for that class.


(4) $$\text{mAP}=\frac{1}{N}\sum_{i=1}^{N}{\text{AP}}_{i}$$

where *N* is the number of classes and AP*_i_* is the average precision for the *i*-th class.

These metrics are computed for each dataset and summarized to assess the overall performance of the YOLOv8l model.

### 4.4 Empirical findings

**Table 4** presents the performance evaluation of various YOLOv8 model variants on the Kasaisa dataset.

**Table 4:** Performance evaluation of YOLOv8 model variants on the Kasaisa dataset.

Model	Class	Instances	P	R	mAP	Inference
YOLOv8n	Healthy	10	0.871	0.7	0.806	228.6
	Infected	18	0.764	0.444	0.535	
	All	28	0.817	0.572	0.67	
YOLOv8s	Healthy	10	0.654	0.8	0.733	316.8
	Infected	18	0.435	0.778	0.643	
	All	28	0.544	0.789	0.688	
YOLOv8m	Healthy	10	0.876	0.6	0.828	615.3
	Infected	18	0.791	0.444	0.625	
	All	28	0.834	0.522	0.727	
YOLOv8l	Healthy	10	0.729	0.9	0.873	1072.5
	Infected	18	0.53	0.556	0.602	
	All	28	0.629	0.728	0.737	
YOLOv8x	Healthy	10	0.586	1	0.858	1447.1
	Infected	18	0.728	0.3	0.621	
	All	28	0.657	0.65	0.74	

YOLOv8l stands out as the optimal choice due to its balanced trade-off between accuracy and inference speed. It achieves a high mAP of 0.737, surpassing all other variants except YOLOv8x, while maintaining a manageable inference time of 1072.5 ms, which is significantly lower than that of YOLOv8x (1447.1 ms). Furthermore, it delivers consistent results across all classes, with both precision and recall exceeding 0.52 for healthy and infected categories. In contrast, the other variants have at least one class falling below this threshold. Thus, YOLOv8l is the preferred model for this task.

### 4.5 Experimental results

The following sections present the experimental results, highlighting the performance of the proposed model across different datasets, each reflecting various conditions.

#### 4.5.1 Performance analysis on multiple datasets

The results presented in **Table 5** provide a comprehensive performance evaluation of the YOLOv8l model across the Kasaisa, Dikumari, and Kukareta datasets.

**Table 5:** Performance evaluation of the YOLOv8l model on the Kasaisa, Dikumari, and Kukareta Datasets.

Dataset	Class	Instances	P	R	mAP
Kasaisa	Healthy	10	0.729	0.9	0.873
	Infected	18	0.53	0.556	0.602
	All	28	0.629	0.728	0.737
Dikumari	Healthy	9	0.819	0.889	0.822
	Infected	11	0.695	0.273	0.406
	All	20	0.757	0.581	0.614
Kukareta	Healthy	20	0.747	0.75	0.825
	Infected	9	0.73	0.556	0.64
	All	29	0.738	0.653	0.733

The model achieves comparable accuracy, as indicated by the mAP, on the Kukareta and Kasaisa datasets, with scores of 0.733 and 0.737, respectively. In contrast, the Dikumari dataset shows a lower mAP of 0.614, which may be attributed to the subtle changes in the infected leaves within this dataset. These subtle variations can make it challenging for the model to detect infections, sometimes posing difficulties even for the human eye.

In terms of processing efficiency, **Table 6** highlights the speed metrics associated with each dataset.

**Table 6:** Speed metrics for the Kasaisa, Dikumari, and Kukareta datasets.

Dataset	Preprocess (ms)	Inference (ms)	Postprocess/Image (ms)
Kasaisa	1.1	1072.5	0.4
Dikumari	1.0	1072.2	0.3
Kukareta	1.4	1018.4	0.4

All times for each processing stage are reported in milliseconds (ms).

The YOLOv8l model exhibits rapid preprocessing times across all datasets, with the Kukareta dataset demonstrating the fastest inference time at 1018.4 *ms*. However, the Kasaisa and Dikumari dataset exhibits a significantly longer inference time (> 1072 *ms*), likely due to the complexity of detecting subtle infections. The post-processing times are relatively low for all datasets, indicating that the model’s pipeline is efficient overall.

For a more detailed analysis of the model’s training performance, the loss graphs for box and mask loss are presented in **Supplementary Figures 2-4**.

**Figure 8:**
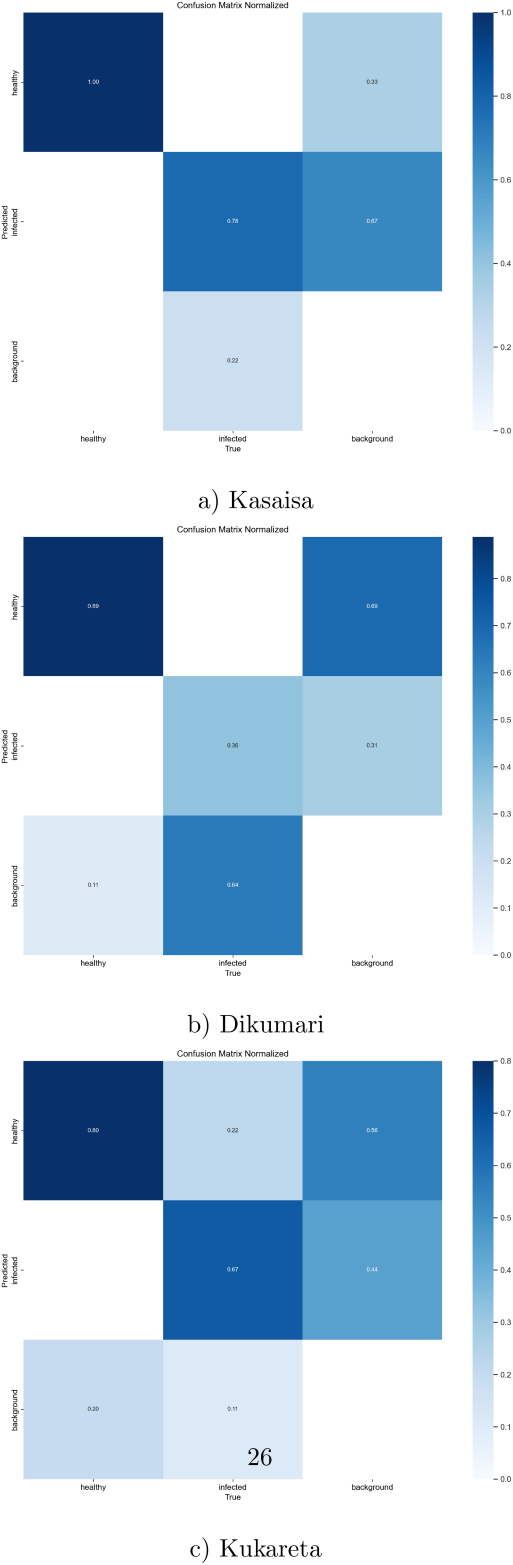
Normalized confusion matrix from **(a)** Kasaisa, **(b)** Dikumari, and **(c)** Kukareta farms, showing classification results of healthy and unhealthy tomato leaves from the TomatoEbola dataset.

Additionally, the confusion matrix for the YOLOv8l model is shown in **Figure 8**, providing insights into the model’s classification performance across different classes.

Overall, the findings suggest that the YOLOv8l model is effective in detecting plant diseases, with certain datasets posing more challenges than others. The combination of high accuracy and efficient processing times demonstrates the model’s potential for real-world applications in agricultural disease detection.

#### 4.5.2 Sample prediction outcomes

**Figure 9:**
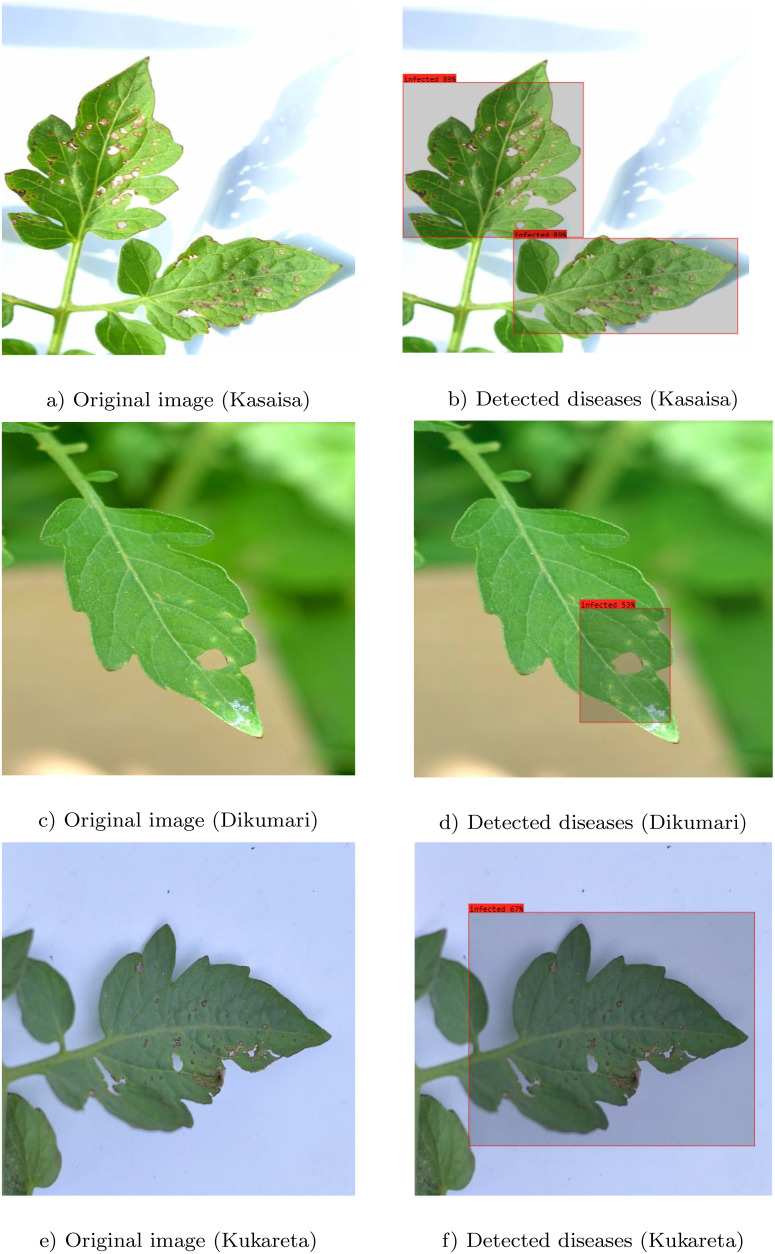
Original images **(a, c, e)** and detected **(b, d, f)** regions of diseases using the YOLOv8 large model. Each pair illustrates the identified infected areas in images from the TomatoEbola dataset.

The results depicted in **Figure 9** clearly illustrate the effectiveness of the proposed method in accurately identifying infected areas in images from the TomatoEbola dataset. Each pair of original and detected images demonstrates the model’s ability to highlight the specific regions affected by disease, providing a visual confirmation of its detection capabilities.

Moreover, the accurate delineation of infected regions across both datasets reinforces the robustness of the YOLOv8 large model. This level of precision is crucial for timely and effective disease management in agricultural practices, enabling farmers to make informed decisions based on the model’s predictions. Overall, the figure highlights the method’s reliability in detecting plant diseases, contributing significantly to advancements in agricultural diagnostics.

#### 4.5.3 Comparison with state-of-the-art object detection models

Here, results are presented with upper and lower bound of 95% confidence interval obtained from multiple independent test runs.

**Table 7** presents a comparison of the proposed approach with state-of-the-art methods, specifically Faster-RCNN, YOLOv5l, and YOLOv8n, across three subsets of the TomatoEbola dataset (Kasaisa, Dikumari, and Kukareta).

**Table 7:** Comparison of the proposed approach with other state-of-the-art methods.

Model	mAP		
	Kasaisa	Dikumari	Kukareta
Faster-RCNN	0.614	0.57	0.685
YOLOv5l	0.57	0.536	0.658
YOLOv8n	0.67	0.585	0.684
Proposed	**0.737** ± 0.095**	**0.614** ± 0.009**	**0.733** ± 0.02*

** represents *p <* 0.001 and * represents *p <* 0.005.

Note bold highlights indicate the best performance for each dataset.

As can be seen, the results indicate that the proposed approach achieves higher performance compared to the state-of-the-art methods across all three TomatoEbola datasets, suggesting that the method demonstrates more stable performance across different datasets, further strengthening its robustness.

The differences in performance are statistically significant, as evidenced by a two-sample t-test (all p <.005), indicating that the proposed method is not only robust but also dependable for practical applications, highlighting its effectiveness in detecting tomato leaf diseases. This makes it a more reliable choice than the existing state-of-the-art models.

#### 4.5.4 Comparison with similar studies

The results presented in **Table 8** demonstrate that although this is not a direct comparison, the proposed approach has shown a marked improvement over other methods in predicting plant diseases, including those affecting tomato leaves.

**Table 8:** Comparison of the proposed approach with similar studies conducted in detecting plant leaf disease.

Reference	Dataset	Plant	Model	mAP
(Giakoumoglou et al., 2023)	Collected	Tomato	Faster R-CNN	0.58
(Shill and Rahman, 2021)	PlantDoc	Apple, Bell pepper, Blueberry Cherry, Corn, Grape, Peach, Potato, Raspberry, Soybean, Squash, Strawberry, Tomato	YOLOv3	0.53
(Shill and Rahman, 2021)	PlantDoc	Apple, Bell pepper, Blueberry Cherry, Corn, Grape, Peach, Potato, Raspberry, Soybean, Squash, Strawberry, Tomato	YOLOv4	0.55
(Liu and Wang, 2020b)	PlantVillage	Tomato	Faster R-CNN	0.56
Proposed	TomatoEbola	Tomato	YOLOv8	0.737

This indicates that the methodologies employed in the proposed framework are effective in enhancing detection accuracy and performance. These findings highlight the robustness and superiority of the proposed method, making it a valuable tool for accurate and efficient tomato leaf disease detection.

#### 4.5.5 Overall impact

In contrast to traditional approaches, such as widespread pesticide application, the AI-driven solution offers a more sustainable and targeted method for managing pests like Tuta absoluta. By enabling precise detection of affected areas, farmers can apply pesticides only to the infested regions or remove damaged crops before the pests spread and cause further harm. This reduces the excessive use of chemicals, minimizing environmental impact and lowering production costs. The ability to identify infestations early allows for timely interventions, leading to more efficient pest management and improved crop health without the drawbacks of blanket pesticide use.

## 5 Discussion

This paper proposed an innovative approach for detecting Tuta absoluta tomato leaf diseases on the TomatoEbola dataset. We created annotations by drawing bounding boxes to label both infected and healthy plant leaves within the dataset. The performance of the proposed approach, based on YOLOv8l, was assessed to investigate its recognition speed and detection efficacy.

Our findings indicate that the YOLOv8l algorithm is effective in detecting both infected and healthy tomato leaves, achieving a mean average precision of nearly 74% and a fast inference time, averaging less than 1055 *ms* across all datasets. These results demonstrate the effectiveness of the proposed method and its potential for deployment in real-world applications, given its success in maintaining both accuracy and speed.

The results demonstrate that the proposed approach consistently outperforms state-of-the-art methods, including Faster R-CNN, YOLOv5l, and YOLOv8n, across all subsets of the TomatoEbola dataset. This superior performance, coupled with the method’s stability across different datasets, underscores its robustness in detecting tomato leaf diseases. Furthermore, statistical significance analysis using a two-sample t-test (see Section 4.5.3) confirms that the observed improvements are not due to random variations but rather to the effectiveness of the proposed model. These findings suggest that the method is not only more accurate but also more reliable for real-world applications, where precise and efficient detection of plant diseases is critical for timely intervention and improved agricultural outcomes.

However, it is noteworthy that the approach achieved the lowest mean average precision on the Dikumari dataset. This discrepancy can be attributed to the subtle nature of the disease images captured from the Dikumari farm, which poses challenges for detection. Consequently, this resulted in a higher inference time for this dataset, reflecting over a 620% increase compared to the Kasaisa and Kukareta datasets.

Despite these challenges, the model’s ability to achieve a higher mean average precision of up to 0.737 compared to similar studies (Liu and Wang, 2020b; Shill and Rahman, 2021; Shill and Rahman, 2021; Giakoumoglou et al., 2023) – where all other models recorded below 0.59 suggests its competitiveness within the agricultural application landscape. Moreover, when compared to the benchmark model on the COCO dataset (Roboflow, 2024b), which achieved a mean average precision of 0.529, the YOLOv8l model demonstrates superior performance. This suggests that our approach is not only effective in detecting Tuta absoluta diseases but can also be applied to other crops in the agricultural domain, contributing to advancements in agricultural machine learning applications.

In this study, we opted to utilize the YOLOv8l model for this task, primarily because the TomatoEbola dataset contains a considerably smaller number of images per farm (less than 320 images). This lower image count is particularly suited for the YOLOv8l model, which is designed to be lightweight and efficient, making it suitable for scenarios where computational resources are limited or when working with smaller datasets.

In this study, we leveraged the YOLOv8l model due to the inherent limitations of the TomatoEbola dataset. With fewer than 320 images from all the three farms combined, the dataset presents a challenge for larger, data-hungry models, which are prone to overfitting with limited training examples. The YOLOv8l model, known for its efficiency and smaller footprint, offers a compelling solution. Its deeper architecture allows for better feature extraction, enabling the model to capture subtle disease patterns while maintaining computational feasibility. This ensures robust performance even with a constrained amount of training data, making it an optimal choice for this study.

Overall, the findings from this study suggest the effectiveness of the YOLOv8 large model in accurately detecting Tuta absoluta diseases, providing a foundation for further exploration in agricultural disease detection systems.

However, this study has some limitations. First, the YOLO model is a deep learning architecture that requires a significant amount of data; however, the TomatoEbola dataset contains a relatively small number of images. Future work should focus on collecting additional data to enhance the sample size from each dataset. Additionally, data augmentation techniques, such as generative adversarial networks and other synthetic data generation methods, can be explored to increase both the sample size and diversity, thereby improving the model’s generalizability.

Second, our investigation was limited to the effectiveness of the YOLOv8n model. It remains unclear whether alternative YOLO models could achieve higher accuracy with this data. Future studies should explore the performance of other YOLO variants to fullyassess their capabilities in detecting Tuta absoluta and other agricultural diseases.

## 6 Conclusion

This study demonstrates the effectiveness of AI-driven solutions, particularly the YOLOv8l model, for detecting tomato leaf diseases caused by Tuta absoluta. We successfully annotated the TomatoEbola dataset, creating a valuable resource for ongoing research and applications in agricultural pest management. By leveraging advanced deep learning techniques, we assessed the YOLOv8l model’s performance in real-world scenarios, highlighting AI’s potential to provide sustainable alternatives to traditional pestcontrol methods. This will allow farmers to implement interventions more selectively, minimizing the use of pesticides and enhancing overall effectiveness. Our findings emphasize the critical role of timely detection in mitigating agricultural losses, ultimately contributing to improved food security.

This work paves the way for further exploration of AI applications in agriculture, highlighting the need for continued innovation in addressing the challenges posed by pests and diseases in crop production. Future advancements will benefit from interdisciplinary collaboration between AI researchers and agricultural experts to refine detection models, ensure practical deployment, and develop integrative solutions that align with farmers’ needs and agricultural best practices.

## Data Availability

The datasets presented in this study are available on Zenodo at: https://zenodo.org/records/13324917.

## Footnotes

1 ArcGIS (Esri, 2024) is a geographic information system that enables mapping, analysis, and visualization of spatial data, facilitating the creation of detailed maps and spatial analyses.
