# Supplementary material for: YOLO for early detection and management of Tuta absoluta-induced tomato leaf diseases
Source: Front Plant Sci. 2025 May 20;16:1524630. doi: 10.3389/fpls.2025.1524630 (PMC12130032; doi:10.3389/fpls.2025.1524630)
Supplement: Supplementary file 1 [file DataSheet1.pdf]

1. Supplementary Images

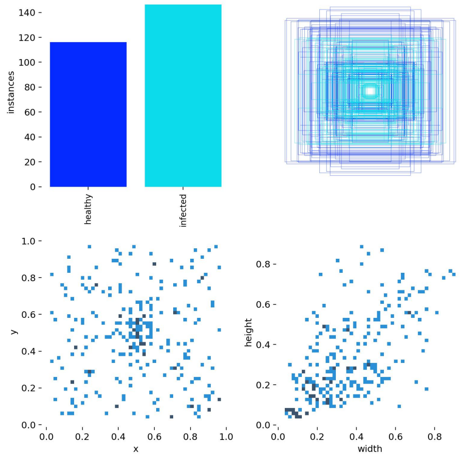

a) Kasaisa

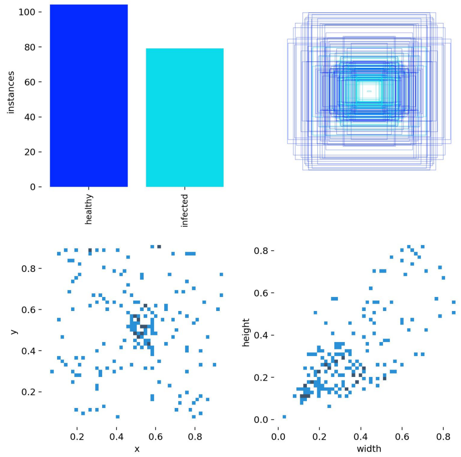

b) Dikumari

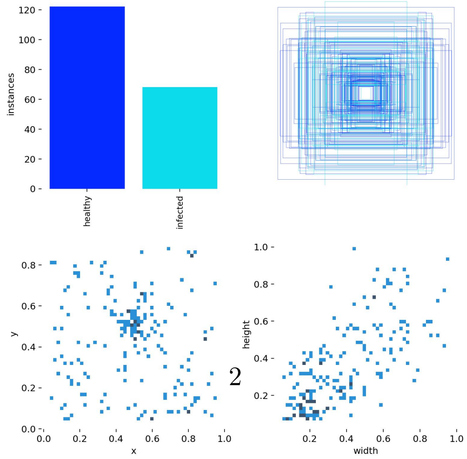

c) Kukareta

Figure 1: Statistical analysis of bounding box labels in the datasets from (a) Kasaisa, (b) Dikumari, and (c) Kukareta. In all figures, top-right: number of labels; top-left: distribution

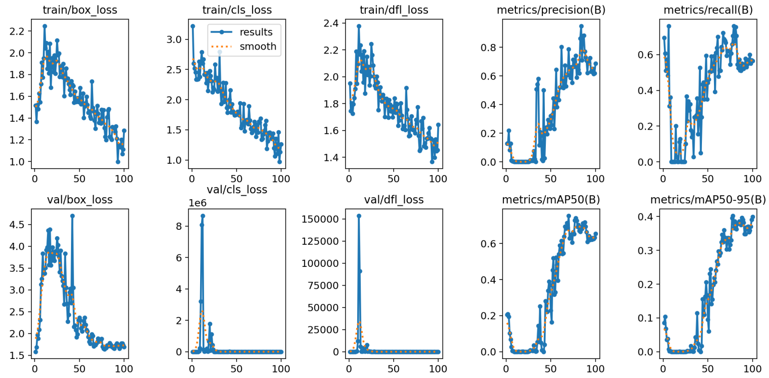

Figure 2: Box and mask loss graphs for the training and validation sets of the YOLOv8l model from the Kasaisa farm.

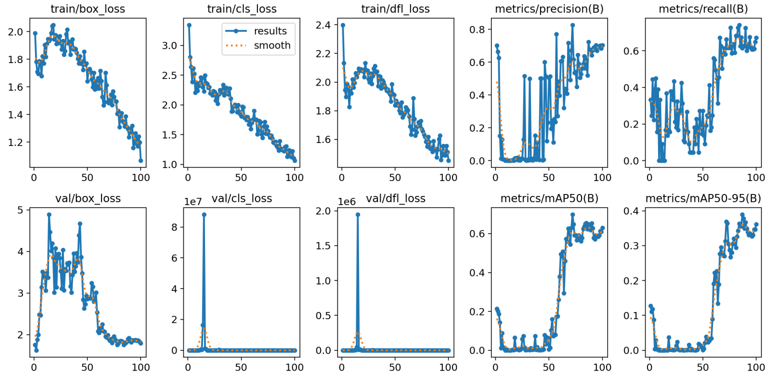

Figure 3: Box and mask loss graphs for the training and validation sets of the YOLOv8l model from the Dikumari farm.

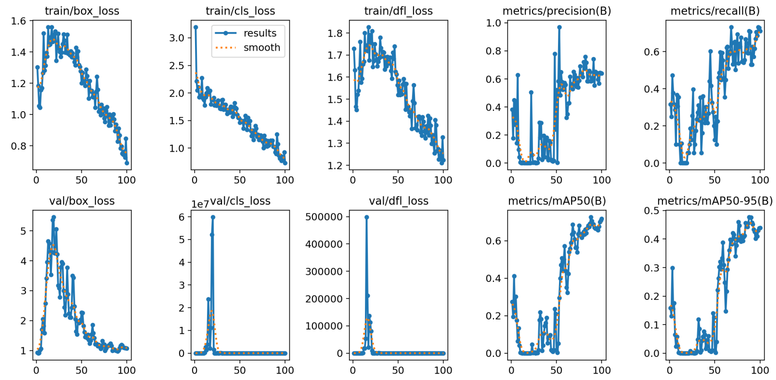

Figure 4: Box and mask loss graphs for the training and validation sets of the YOLOv8l model from the Kukareta farm.
